# Lived experiences of Iranian prehospital emergency technicians in facing women’s emergencies: a phenomenological study

**DOI:** 10.1186/s12873-024-01019-5

**Published:** 2024-06-10

**Authors:** Milad Ahmadi Marzaleh, Mahmoudreza Peyravi, Esmaeil Ahmadi, Hadi Mahmoodi, Iman Shakibkhah, Hossein Armin

**Affiliations:** Department of Health in Disasters and Emergencies, School of Health Management and Information Sciences, Shiraz University of Medical Sciences, Shiraz, Iran

**Keywords:** Prehospital emergencies, Women, Lived experiences, Taboo, Health

## Abstract

**Introduction:**

: This study aimed to investigate the work problems and challenges of male prehospital emergency technicians when faced with female medical emergencies. Given that qualitative research has not been done in this field, planning to find the weak points and improve the quality of prehospital emergency as the first line of treatment for female emergency patients, which is considered an important part of the health care system, is considered important and valuable. Therefore, this phenomenological study was conducted in 2023.

**Methods:**

This study was conducted using a qualitative method of phenomenology in 2023. The environment of the research was urban and road prehospital emergency centers in Iran and the data were collected through interviews with EMS technicians. The collected data were analyzed using Smith’s approach to explain the lived experiences of EMS technicians facing female emergencies or women’s emergencies in Iran.

**Results:**

All the 15 participants were men. Their mean age was 35 years, with a range of 25 to 45 years, and with a mean work experience of 10.54 years with a range of 4 to 20 years. The lived experiences of 115 emergency technicians in facing women’s emergencies in Iran were placed in four main themes cultural-social factors, organizational factors, human resources-related factors, and administrative-legal factors.

**Conclusion:**

EMS personnel face various obstacles in carrying out missions related to women’s emergencies, Considering the critical nature of women’s emergencies, it is recommended that policymakers and clinical educators improve the level of community culture, communication skills, theoretical and practical training, respecting privacy, hiring female personnel, adding specialized equipment, amending and changing laws, removing road-traffic obstacles and to support personnel, patients and their families psychologically to optimize performance in women’s emergencies.

## Introduction

Emergency medical services (EMS) are the main components of providing significant health care to help reduce out-of-hospital mortality [1]. Similarly, they are strongly recommended and supported by the World Health Organization (WHO), which gives such services a role in both the healthcare system and the protection of the individual’s right to access the healthcare [2]. Prehospital emergencies play a vital role in providing timely care to patients [3]. Today, in most societies, quality prehospital emergency care is an essential part of caring for patients in need of emergency care [4]. To provide care in complex, unsafe, and uncontrollable situations in various environments such as home locations, public places, and crime and accident scenes, EMS workers are needed [5].

Prehospital obstetric events encountered by EMS can be risky [6]. Postpartum hemorrhage (PPH) is a serious obstetric emergency and one of the five main causes of maternal mortality worldwide [7]. Symptoms such as spotting or bleeding, amniotic fluid leakage, abdominal pain, or back pain may indicate a miscarriage [8]. However, even during the normal course of pregnancy, warning signs and obstetric complications may occur, which are usually unpredictable and require appropriate attention [9]. Due to the possibility of pregnancy-related health complications, specialized attention, including tests or evaluations in additional consultations to monitor their condition is necessary in the case of risk detection [10]. Providing timely and quality emergency obstetric care by a skilled healthcare professional and functional referral systems can reduce maternal complications and mortality [11]. Warning signs during pregnancy include vaginal bleeding, abdominal pain, swelling, headache, fever, or specific changes in fetal movement. These symptoms may be associated with a risk to the mother’s or fetus’s health and life and are generally unexpected [12]. Significantly, such things and other cases and warning signs may occur at any time during pregnancy and are a threat to the woman’s health and life [8].

Pregnant women expect necessary measures to ensure the provision of the best professional medical care during this critical period [13]. To ensure both mother and child’s safety, WHO emphasizes the importance of skilled attendants assisting laboring women [14]. The issue of decreasing the death rate of mothers and children under the age of five has always been one of the major goals of all human health activities both internationally and locally [15]. Not only should a paramedic’s profession enjoy great social trust, but also it should enjoy people’s awareness of the competencies and qualifications of their representatives [16]. Every person working in the EMS system should have extensive knowledge and skills at a high level since random events do not allow planned patient selection to the medical personnel [17]. EMS and prehospital care providers have the potential to significantly improve obstetric patient outcomes through timely prehospital medical interventions and transition to center-based care [18].

The majority of EMS personnel in Iran are male, so the gender-based challenge in delivering and receiving care, particularly in the most private and intimate conditions, can be one of the most important challenges. Even though, in some childbirth missions, midwives are also dispatched along with personnel for medical emergencies, it is not always possible, especially during evening and night shifts and in rural areas. Therefore, the personnel of medical emergencies are mostly dispatched to such missions without the attendance of a midwife or a female healthcare professional [19]. The EMS providers in Iran consist of a combination of nurses and EMTs (Emergency Medical Technicians). The system’s missions are carried out by two EMS providers [20]. Moreover, currently, only males are employed in pre-hospital emergency care and there is not any female staff [4].

Many male emergency medical service personnel do not feel ready or even are afraid of managing the prehospital care for a pregnant patient due to their limited experience with these female patients [6]. For example, many specialistic gynecological examinations cannot be performed in an ambulance; therefore, a patient requiring diagnosis and observation should go to an appropriate hospital or clinic [21].

The purpose of this study is to investigate the career problems and challenges of male prehospital emergency technicians when faced with medical emergencies involved a female. Given that no qualitative research has been conducted in this field, planning to find the weak points and improve the quality of prehospital emergency as the first line of treatment for female emergency patients, which is considered an important part of the health care system, is considered important and valuable. Therefore, this phenomenological study was conducted in 2023.

## Methods

From June 1, 2023, to August 11, 2023, this study was conducted with a qualitative phenomenological method descriptively rather than interpretively. The environment of the research was urban and road prehospital emergency centers in Iran; data were collected through interviews with EMS technicians and analyzed using Smith’s Approach to explain the lived experiences of EMS technicians in dealing with women’s emergencies in Iran.

### Participants

Semi-structured in-depth interviews were conducted with EMS technicians. The criteria for participants to enter the interview included having at least 4 years of work experience in prehospital emergency, having at least an associate degree in medical emergency, and having faced women’s emergencies. The exclusion criterion also included participant’s unwillingness to participate in the study.

The sampling method was purposive. People who were rich in the mentioned information were identified. Then, by explaining the objectives and importance of the research to these people and obtaining their consent, as well as the researcher’s visit at the appropriate time, an interview was conducted with them. The numbers #1, #2, and … were used to name people. People’s names were not entered in the demographic forms.

### Data collection

Deep and semi-structured questions were asked regarding the technicians’ experiences in dealing with women’s medical emergencies. In-depth questions included: “What have been your lived experiences in dealing with women’s medical emergencies?” and “Please mention examples of your experiences in this regard.” Based on the answers given by the participants, follow-up questions were also asked. At the end of each interview, the participant was asked to mention any experience related to the purpose of the study that wasn’t asked, if they wanted. The interview lasted from 31 to 65 minutes. The interviews were conducted in a quiet place. After data collection, the interviews were concluded. Follow-up questions, such as: “How?“, “Why?“, “Can you explain more about …?” were also asked during the interview. Also, while talking about the topic under discussion, we examined many other aspects of the opinions of the interviewees that were raised during the conversation. 15 interviews were conducted with 15 participants. Finally, due to data saturation and repetition of the codes, the interviews were terminated. In order to avoid possible problems in recording the voice of the interviewees, the interviews were recorded by 2 tape recorders, and they were implemented immediately after they were done.

### Data analysis

Simultaneous with the data collection, analysis was also done. All interviews were transcribed word by word immediately after recording. The researchers also took notes during the interview. After conducting each interview, first, its script was written down and reviewed several times to get a general understanding of it. An interpretative summary was written for each of the interview scripts, and an attempt was made to understand and extract the meanings hidden in it. Data analysis was done using Smith et al.‘s approach, which was based on Heidegger’s phenomenology and had a team nature; its step-by-step stages are as follows: **(1) Analyzing the data**: This phase was done to understand the data. The researcher immersed himself in the data and re-read the data and information several times. Analysis by Smith’s method included the following steps: **A**- Initial encounter: reading and re-reading the text of the interviews and taking notes. **B**- Identifying and labeling the themes: In this part, the extracted codes were labeled with the desired theme (essence or theme) with professional terms. **C**- Listing and clustering of themes: In this stage, the organization of the extracted codes, classes, subclasses, sub-subclasses, and themes were discussed. **D**- Creating a summary table: A table was designed, and it was done at several levels of classification, like code, subclass, sub-subclass, and finally theme. And in this table, in front of each extracted code, a direct quote from the script of the interviews was mentioned along with the page number and details of the participant. If the codes or themes that were extracted in the previous steps were not suitable, they were removed in this stage. **(2) Combining the items**: In this stage, a comprehensive table was designed and by reading the tables of each participant that were designed in the previous step, the codes and finally the themes were categorized. **(3) Reporting, writing, and describing the phenomenon**: Finally, a full report on each of the themes, codes, subclasses, and sub-subclasses was presented and discussed.

A manual method was used to analyze the data. Likewise, deductive phenomenology (bracketing) was used, which means leaving aside the personal opinions and assumptions of the researcher. A review of the scripts and sources was done after data analysis.

### Rigor

In order to guarantee the validity and accuracy of qualitative data, Guba and Lincoln criteria were utilized, which include validity, transferability, reliability, and verifiability [22], .

### Ethical considerations

The researchers, after obtaining the necessary permits and receiving an introduction letter from the Research Vice-Chancellor of the School of Health Medical and Information Sciences Committee (Shiraz University of Medical Sciences) (Biomedical Research Ethics Committee)( IR.SUMS.NUMIMG.REC.1402.128), presented it to the research samples and introduced themselves to the participants and explained the purpose of the research, making sure that all the things recorded during the interview remained confidential. Afterwards, the participants who were willing to participate in the study were selected. At the same time, the participants were assured that they could leave the interview process if they were not willing to continue at any stage of the research. Other ethical considerations included : **(1)** Obtaining written consent from experts, **(2)** Assuring the participants that the results of the study would be provided to them if they wished, **(3)** Observing the ethical considerations in terms of the confidentiality of information of the research units, **(4)** Thanking and appreciating all the people who cooperated with us in connection with conducting the research, **(5)** Obtaining permits from the ethics committee (Biomedical Research Ethics Committee), and **(6)** Being free to continue the interviews or even participate in the study.

## Results

Participants’ mean age was 35 with a range of 25–45 and with a mean work experience of 10.54 years ranging from 4 to 20 years. The demographic and occupational characteristics of the participants are presented in Table 1.

**Table 1:** Demographic and occupational characteristics of the participants

Variable		Frequency	Percentage
Length of work experience (in years)	4–10 10–15 15–20	8 5 2	53.3 33.4 13.3
Degree of education	Associate degree in medical emergency Bachelor’s degree in medical emergency Bachelor’s degree in nursing	7 4 4	46.6 26.7 26.7
Base type	Urban Road	11 4	73.3 26.7
Marital status	Single Married	3 12	20 80

The 15 personnel of EMS teams who expressed a willingness to participate in the study and had experience in facing the emergencies of women were selected through purposive sampling from urban and road bases of Esfahan, Iran, who had experience of working in prehospital emergency of other cities including Tehran, Shiraz, Shahr-e Kord and Ahvaz in addition to Esfahan and then were interviewed. By analyzing the interviews, a total of 527 codes were extracted. By removing the duplicate codes and merging the similar ones, finally, we found the lived experiences of 115 emergency technicians in facing the emergencies of women in Iran in the four main themes of cultural-social factors, organizational factors, and human resources factors and administrative-legal factors. The sub-themes and sub-sub-themes are also given in Table 2.

**Table 2:** Themes and sub-themes from interviews with participants to explain the lived experiences of EMS technicians in dealing with women’s emergencies in Iran

Main theme	Sub-theme	Sub-sub-theme
	Environmental-social factors	Difficulty in carrying and moving the patient. Road traffic and not giving way to an ambulance. The bumpiness of the streets and alleys The roads are impassable The interference of companions Failure to respect privacy Space creation in the virtual world Being alone (patient, unaccompanied(
	Communication factors	Lack of dialect and verbal communication Client’s suspicion of EMS technician
	Emotional-sentimental factors	EMS technician’s fear of exposure Visualization of uncomfortable scenes in the EMS technician’s mind The distress of the EMS technician The distress of the client The distress of the companions Violence with EMS technician EMS technician stress EMS technician affected
	Ethnic-religious factors	People’s religious prejudices Ethnic traditions
Organizational factors	Lack of equipment	Lack of educational facilities and equipment Lack of medicines and related medical equipment
	Lack of manpower	Lack of female EMS technicians Lack of dedicated women’s EMS base Insufficient number of EMS technicians
	Lack of education	Insufficient theoretical training for EMS technicians Insufficient practical training for EMS technicians
Factors related to human resources	Individual characteristics of human resources	Inappropriate EMS technician attire Inappropriate EMS technician approach and behavior
	Dissatisfaction of personnel	EMS technician discouragement Lack of EMS technician motivation
Administrative-legal factors	Supporting factors	Lack of EMS technician psychological counseling and support Lack of lawyer and EMS technician support representative
	Content factors	Inadequate rules Contradiction in relevant laws

A summary of what the technicians said is given below:

Participant #2 said: *“Unfortunately, the culture has not yet developed in such a way that people are very comfortable with the emergency personnel unless we ourselves ask a lot of questions so that if there is a problem related to women’s disease and they tell us. Many times, they hide it at first because they always hide it when we ask about their illness because of other people being around them and they don’t like their family and friends to know. They are always not comfortable with the staff when we are men and they are women and we cannot find out about their illness unless they have to say. Because of their cultural problems, if they have a gynecological disease, they rarely tell the emergency personnel about it, and they do not allow the emergency personnel to observe and perform physical examinations. They are not very comfortable with it.”*

Participant #4 said: *“In case we have cultural problems with our own people, Afghans often cause cultural problems. For example, Afghans are not under the coverage of a doctor. They are poorer financially and have multiple pregnancies and multiple abortions. It doesn’t matter much to them either, and these pregnancies increase the possibility of us dealing with such cases. Also, because we are dealing with women and the emergency personnel are all men, this is not tangible for them. Of course, the Iranians themselves do not accept it until they are in critical conditions and their wives and children are not in danger. Cultural problems are such things.”*

Participant #5 said: *“For example, we have a lot of Afghans here in Iran, but they don’t really understand what we say or what we want from them. Once, when I was in the south, they were talking in the Achumi dialect (an accent in the city of Bandar Abbas) and I couldn’t understand it. Once I saw that the work was going on and the lady was giving birth in the ambulance. For example, there were different dialects that we could not communicate with them, and we encountered problems.”*

Participant #7 said: *“Mostly, people’s emotions and feelings are provoked, you know; for example, we are provoked, but we act like we have come to calm them down. If we want to be like them, we can’t really control them anymore. We went for abortions and this. Abortion is a difficult thing that has a very negative effect on the person and her family. However, if we don’t manage it, their behavior will get worse; we will be affected because a person’s life has been lost. “*

Participant #1 said: *“Our equipment is average, good, but it could be a little more helpful if they can employ a female technician or midwife; now, it is better to talk about the round-the-clock standby shift, or the hospital should plan to have a midwife as a consultant. Now, ambulances are limited, I mean, they cannot have advanced equipment, but there is basic equipment and there can be more equipment like in developed countries and other places and it is not enough to have two people in the emergency room; if there is a question of resuscitation, it is a very difficult task, and we have to think about giving birth to a child. Also, we have to think about the pregnant woman and should take her to a medical center immediately; if the equipment and facilities were more up-to-date like in other countries, we could be more responsive.”*

Participant #10 said: *“In my opinion, there is little education about women. In other cases, such as cardiac emergencies and trauma, they hold classes and bring models. They have long-term training and take exams, but for women’s emergencies, because they cannot have a personal model, even a person who has just entered a women’s emergency, the courses he attends are all theoretical, and until he sees a pregnant woman or a case of abortion, how can he want to practically give birth to a child? When to remove the umbilical cord? Where to take and give birth to a child? If it was possible, there was a place where he could be trained in these subjects or could go to a maternity hospital or a hospital where he could experience a case or see cases of bleeding, he could have done a much better job and should be trained in this field.”*

Participant # 6 said: *“We are not protected by some rules, like other countries, but by talking and behaving well, we can work again in these conditions, and people trust us and let us do the work; unfortunately, the way some personnel dress and behave is such that in some places they don’t even allow us to enter the house, even if we want to do work, so the appearance has a great impact.”*

Participant #12 said: *“Sometimes, they condemn a person that he should have done it; sometimes, when you do it, they ask, ‘why you did so?’ There is a contradiction in their laws; there is no specific law that one can know for sure, whether one can come to this border or not, you are going to get into trouble somewhere; they say ‘You should have done it medically, why didn’t you do it?’ If you do it once, they will condemn you again, and say ‘why did you do it? Why did you check? Shouldn’t you have been examined?’ There is no specific law at all, we don’t have a correct law, and we are not supported anywhere.”*

## Discussion

This study was conducted with the goal of investigating the challenges and problems faced by the EMS technicians of urban and road bases in Iran when encountering women’s emergencies in general (types of women’s problems), and it was shown that there were many challenges in the field; they were related to cultural, social, environmental, relational, emotional, organizational, human resources, administrative, and legal factors. Problems such as lack of etiquette, lack of sufficient recognition of EMS in the community, difficulty in transporting and moving the patient, traffic and not giving way to the ambulance, bumpiness of alleys and streets, impassibility of roads, interference of companions, lack of respect for privacy, creation of space in the virtual world, lack of medical equipment and supplies, lack of manpower, dissatisfaction of the personnel, and lack of psychological support of EMS, which were found in the results of this study. It is in line with the findings of a qualitative study in 2015 entitled “Challenges of prehospital emergency in Mashhad” [23]. Also, in another qualitative study conducted in Iran in 2018 entitled “Qualitative experiences of EMS personnel in ethical decision-making“ [24], is the results were in line with the cultural, social, and organizational challenges of the present study. When EMS personnel face with ethical challenges, they can make an ethical decision in the least amount of time in emergency situations [17].

In dealing with ethical challenges, the personnel are affected by various factors such as paying attention to the patient’s values ​​and wishes, professional and organizational values, socioeconomic factors, and cultural factors, as well as the boundaries of the therapeutic relationship with the health team and the patient [17]. Also, in a study on the challenges of locating emergency scenes of the EMS system, which was ousted in 2023 in Rwanda in Central Africa [25], the lack of manpower was also mentioned. The theoretical and practical EMS training problems found in the present study are also in line with those in the study conducted in 2021 in the United States of America, which pointed out the insufficient experience of EMS technicians in dealing with women’s emergencies. Many EMS personnel of emergency medical services do not feel prepared or are even afraid to manage the prehospital care of a pregnant patient due to their limited exposure to these female patients [6].

Although in that study, one case of women’s emergencies, which is pregnancy, is mentioned, in the present study, the issue of women’s emergencies in general (types of women’s emergencies) was studied, which is unique in its type. The comparison of studies shows that some problems in the prehospital emergency are general and not only related to women’s emergencies; although in the transportation and transfer of patients with women’s emergencies, especially pregnant patients, equipment, medical supplies, special drugs, and the protocols and laws related to women’s emergencies have special importance and place according to the cultural context and the ethnic and religious prejudices of the society, similar researches were not found only for women’s emergencies in general, so that the results of the present study could be compared with them in detail and more specifically. The EMS technician’s fear of exposure, visualization of uncomfortable scenes in the EMS technician’s mind, the EMS technicians’ distress, the client and her companions’ distress, as well as the technician who is affected are the emotional-sentimental factors which lead to an increase in stress of EMS technicians in dealing with female emergencies, which have a negative impact on their jobs. This result is in line with that of the study conducted in 2016 entitled “Relationship between occupational stress and the quality of work life of prehospital emergency workers in Shiraz“ [26].

One of the problems was the lack of dialect and verbal communication, which was in line with a study conducted in Kuwait in 2019, which mentioned the language and its consequences on the quality of work of EMS technicians [27]. Because language differences affect prehospital service providers and the quality of their work [20], EMS technicians face challenges when providing medical services and when communicating with their patients. Therefore, unknown issues that can potentially improve prehospital performance should be addressed [20].

Regarding the clients’ suspicion, it should be mentioned that some people’s distrust in male EMS technicians and even lack of trust in their skills, concealment of gynecological diseases in some clients, and insufficient information in suspicious cases are among the other problems found in this study. It also stems from the culture, religious prejudices, and ethnic traditions of the people. Lack of female EMS technicians, lack of dedicated EMS women’s station, inappropriate EMS technician attire and inappropriate EMS approach and behavior, EMS technician discouragement, EMS technician demotivation, which is one of the problems related to human resources, as well as lack of counseling and psychological support, lack of lawyer and EMS technician support representative, insufficient laws, and contradiction in the relevant laws, which is one of the legal-administrative factors are the sources of problem.

In a qualitative study conducted in 2012 on prehospital emergency technicians in Golestan province [28], the problems related to organizational factors, administrative rules and regulations, educational programs, facilities, equipment, and required human resources, which are in line with some of the problems of the present study, were pointed out. However, in that study, the problems related to women’s emergencies were not specifically investigated, but in general, because the technicians’ encounter with women’s emergencies requires their own training programs and rules and regulations, further research is required. To solve these problems, it is necessary to reform the organizational structure, revise administrative laws and regulations, review educational programs, provide facilities and equipment and human resources, reform the monitoring and evaluation system of employees, and provide welfare facilities for the personnel. Therefore, these steps should be taken in order to improve the quality of prehospital emergency services through providing education to people and cooperation and participation of other organizations including the media and radio and television at the social level.

Also, in a qualitative study carried out in Iran in 2019 [29], it has been highlighted that because prehospital decision-making is a challenging process and it is necessary to identify this process [22]. Some of the findings of this study, which are in line with those of the present study, showed that some technicians faced fear and anxiety caused by unclear and ambiguous tasks, insufficient authority, insufficient qualifications, and shortcomings in medical protocols. These factors are caused by insufficient trust, insufficient supervision, and lack of protective laws [22]. Therefore, the technicians are afraid of the legal consequences caused by the possible threat to the safety and dissatisfaction of the patients, which requires continuous training plans for both their knowledge and especially their skills to improve the technicians’ competence [22]. In this study, like previous studies, prehospital technicians face general emergencies, not only women’s emergencies.

As other findings of this study, the interference of people around, ordinary people, and threats and violence against emergency personnel were identified as negative and stressful aspects [22], which were also found in the results of the present study. The reason for people’s interference was their lack of knowledge about the duties and importance of technicians’ prehospital care [22]. In the findings of the study, most of the technicians were dissatisfied with the weak support and follow-up of their organization [22]. The above problems in total cause discouragement, lack of motivation, and increased job stress in prehospital emergency technicians.

If the stress caused by the job is too much, it can cause physical, mental, and behavioral complications for the person, endangering his health. Also, these pressures can reduce the quality of the individual’s work by threatening organizational goals [19], which is an obstacle to providing quality medical services. Because today, in most societies, quality prehospital emergency care is an essential part of caring for patients in need of emergency care [4], these problems can be overcome with proper policymaking, managing and planning at a high level. It is suggested that a similar study should be conducted in other provinces of the country. The results of our study show that it is necessary to conduct more research in the field of women’s emergencies in different regions and provinces with greater cultural and ethnic diversity for a better understanding of the subject. In addition, research should be done in the field of EMS teamwork to provide better medical care by those responsible for providing medical assistance in the face of women’s emergencies.

One of the potential limitations of the current study was that this study was conducted in the context of Iran, which cannot be generalized to countries with different cultural context,

but it seems that due to the common problems, the results can be applicable in low and average-income countries and those with similar cultural contexts. Another limitation of the present study was the use of a minimum associate degree in emergency medicine, a work experience of 4 years and above, and only operational personnel. Therefore, the results may not be generalizable to lower levels of associates, including first responders, administrative, and staff personnel.

## Conclusion

The results showed that emergency medical personnel face various obstacles in carrying out missions related to women’s emergencies, which pose challenges such as social, organizational, human resources, administrative, and legal, especially cultural, both for personnel and for patients involved in women’s emergencies. Therefore, based on the factors identified from the real experiences and insights provided by the participants, considering the critical nature of women’s emergencies, it is recommended that policymakers and clinical educators improve the level of community culture in every possible way, improve communication skills, improving the level of theoretical and practical training, respecting privacy, hiring female personnel, adding specialized equipment, amending and changing laws, removing road-traffic obstacles and to motivate the personnel and provide emotional and psychological support to them, patients and their families to optimize performance in women’s emergencies.

## Data Availability

The data that support the findings of this study are available from the corresponding author, upon request.
